# Clinical applications of mesenchymal stem cells

**DOI:** 10.1186/1756-8722-5-19

**Published:** 2012-04-30

**Authors:** Shihua Wang, Xuebin Qu, Robert Chunhua Zhao

**Affiliations:** 1 Institute of Basic Medical Sciences & School of Basic Medicine, Center of Excellence in Tissue Engineering, Chinese Academy of Medical Sciences & Peking Union Medical College, 5# Dongdansantiao, Beijing, 100005, P.R. China

## Abstract

Mesenchymal stem cells (MSC) have generated a great amount of enthusiasm over the past decade as a novel therapeutic paradigm for a variety of diseases. Currently, MSC based clinical trials have been conducted for at least 12 kinds of pathological conditions, with many completed trials demonstrating the safety and efficacy. This review provides an overview of the recent clinical findings related to MSC therapeutic effects. Roles of MSCs in clinical trials conducted to treat graft-versus-host-disease (GVHD) and cardiovascular diseases are highlighted. Clinical application of MSC are mainly attributed to their important four biological properties- the ability to home to sites of inflammation following tissue injury when injected intravenously; to differentiate into various cell types; to secrete multiple bioactive molecules capable of stimulating recovery of injured cells and inhibiting inflammation and to perform immunomodulatory functions. Here, we will discuss these four properties. Moreover, the issues surrounding clinical grade MSCs and principles for MSC therapeutic approaches are also addressed on the transition of MSCs therapy from bench side to bedside.

## **Introduction**

Stem cells have the capacity to self-renew and to give rise to cells of various lineages. Thus, they represent an important paradigm of cell-based therapy for a variety of diseases. Broadly speaking, there are two main types of stem cells, embryonic and non-embryonic. Embryonic stem cells (ESCs) are derived from the inner cell mass of the blastocyst and can differentiate into cells of all three germ layers. However teratoma formation and ethical controversy hamper their research and clinical application. On the other hand, non-embryonic stem cells, mostly adult stem cells, are already somewhat specialized and have limited differentiation potential. They can be isolated from various tissues and are currently the most commonly used seed cells in regenerative medicine. Recently, another type of non-embryonic stem cells, known as induced pluripotent stem cell (iPSC) has emerged as a major breakthrough in regenerative biology. They are generated through enforced expression of defined transcription factors, which reset the fate of somatic cells to an embryonic stem-cell-like state.

Cellular therapy has evolved quickly over the last decade both at the level of in vitro and in vivo preclinical research and in clinical trials. Embryonic stem cells and non-embryonic stem cells have all been explored as potential therapeutic strategies for a number of diseases. One type of adult stem cells, mesenchymal stem cells, has generated a great amount of interest in the field of regenerative medicine due to their unique biological properties. MSCs were first discovered in 1968 by Friedenstein as an adherent fibroblast-like population in the bone marrow capable of differentiating into bone [1]. It was subsequently shown that MSCs can be isolated from various tissues such as adipose tissue, peripheral blood, umbilical cord and placenta. These cells have a remarkable capacity of extensive in vitro expansion which allows them to rapidly reach the desired number for in vivo therapy [2]. Different laboratories have identified, under partly different isolation or culture conditions, MSCs with specific properties. For better characterization of MSC, in 2006, the International Society of Cellular Therapy defined MSCs by the following three criteria [3]:

(1) MSCs must be adherent to plastic under standard tissue culture conditions;

(2) MSCs must express certain cell surface markers such as CD73, CD90, and CD105, and lack expression of other markers including CD45, CD34, CD14, or CD11b, CD79alpha or CD19 and HLA-DR surface molecules;

(3) MSCs must have the capacity to differentiate into osteoblasts, adipocytes, and chondroblasts under in vitro conditions.

This review will provide an overview of the recent clinical findings related to MSCs. Roles of MSCs in clinical trials conducted to treat GVHD and cardiovascular diseases are highlighted. The therapeutic effects of MSC are mainly attributed to their four important biological properties. Here, we will discuss these four properties and the issues surrounding use of MSCs that need to be addressed during the transition of MSCs therapy from bench side to bedside.

## Clinical applications of MSCs

**Figure 1:**
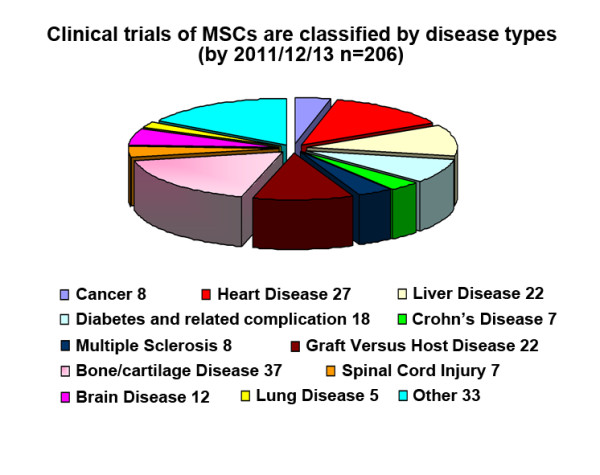
Clinical trials of MSCs are classified by disease types.

**Figure 2:**
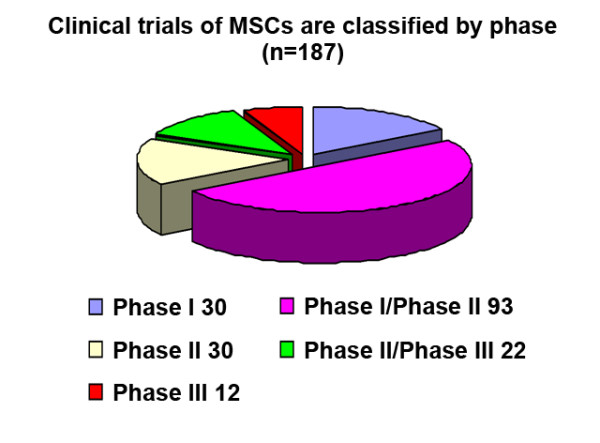
Clinical trials of MSCs are classified by phase.

While accumulating data have shown the therapeutic effects of MSCs in animal models of various diseases, we only focus on the clinical application of MSCs in this review. The first clinical trial using culture-expanded MSCs was carried out in 1995 and 15 patients became the recipients of the autologous cells [4]. Since then, a number of clinical trials have been conducted to test the feasibility and efficacy of MSCs therapy. By 2011/12/12, the public clinical trials database http://clinicaltrials.gov has showed 206 clinical trials using MSCs for a very wide range of therapeutic applications Figure 1). Most of these trials are in Phase I (safety studies), Phase II (proof of concept for efficacy in human patients), or a mixture of PhaseI/II studies. Only a small number of these trials are in Phase III (comparing a newer treatment to the standard or best known treatment) or Phase II /III (Figure 2). In general, MSCs appear to be well-tolerated, with most trials reporting lack of adverse effects in the medium term, although a few showed mild and transient peri-injection effects [5]. In addition, many completed clinical trials have demonstrated the efficacy of MSC infusion for diseases including acute myocardial ischemia (AMI), stroke, liver cirrhosis, amyotrophic lateral sclerosis (ALS) and GVHD.

## MSCs infusion to treat GVHD

Acute graft-versus-host disease (aGVHD) occurs after allogeneic hematopoietic stem cell transplant and is associated with high morbidity and mortality [6-8]. Currently, corticosteroids are the gold standard for initial treatment of aGVHD. However, they are only effective for some patients. Over the past decade, the immunomodulatory functions of MSCs have triggered great interests in their application for GVHD. Le Blanc K et al were the first to transplant haploidentical MSCs in a 9 year old boy with severe treatment-resistant grade IV aGVHD of the gut and liver. They found the clinical response was striking and the patient was well after 1 year [9]. A subsequent study was reported by Ringdén O et al in 2006. They gave MSC to eight patients with steroid-refractory grades III-IV GVHD and one who had extensive chronic GVHD. Acute GVHD disappeared completely in six of eight patients. Complete resolution was seen in gut (6), liver (1) and skin (1). Their survival rate was significantly better than that of 16 control patients. Five patients are still alive between 2 months and 3 years after the transplantation [10]. The beneficial effect of MSCs infusion was then observed in a series of studies (Table 1) .

**Table 1:** A summary of the clinical experience of MSCs in GVHD treatment

**Year of publication**	**Patients (N)**	**MSC source**	**MSC dose**	**outcome**
2007 [11]	6	haplo-identical family donors (n = 2), unrelated mismatched donors (n = 4)	1.0x10(6)/kg	Acute GVHD disappeared completely in five of six patients, four of whom are alive after a median follow-up of 40 months (range, 18–90 months) after the initiation of AMSC therapy. All four surviving patients are in good clinical condition and in remission of their hematological malignancy.
2008 [12]	55	HLA-identical sibling donors (n = 5), haploidentical donors (n = 18), third-party HLA-mismatched donors (n = 69).	1.4x10 (6) (min-max range 0.4-9x10 (6)) cells per kg	30 patients had a complete response and nine showed improvement. No patients had side-effects during or immediately after infusions of mesenchymal stem cells. Three patients had recurrent malignant disease and one developed de-novo acute myeloid leukaemia of recipient origin. Complete responders had lower transplantation-related mortality 1 year after infusion than did patients with partial or no response
2008 [13]	7	hematopoietic stem cell donors (n = 5), third party parental donor (n = 2)	From 0.4x10(6) to 3.0x10(6) per kg based on availability	One out of three patients showed slight improvement of chronic GVHD. Two patients with severe acute GVHD did not progress to cGVHD. One patient received MSC to stabilize graft function after secondary haploidentical transplantation. One patient recovered from trilineage failure due to severe hemophagocytosis.
2009[14]	13	Unrelated HLA disparate donors	A median dosage of 0.9 x 10(6)/kg (range 0.6-1.1).	Two patients (15%) responded and did not require any further escalation of immunosuppressive therapy. Eleven patients received additional salvage immunosuppressive therapy concomitant to further MSC transfusions, and after 28 days, five of them (45%) showed a response. Four patients (31%) are alive after a median follow-up of 257 days, including one patient who initially responded to MSC treatment.
2009 [15]	33	PBSCT combined with MSCs	From 0.5x10 (5) to 1.7x10(6) per kg	Fifteen patients (45.5%) developed grade I–IV acute GVHD (aGVHD) and only 2 (6.1%) developed grade III to IVaGVHD. Nine (31%) of 29 evaluable patients experienced chronic GVHD (cGVHD).
2009 [16]	32	Unrelated, unmatched donor	2 or 8 million MSCs/kg in combination with corticosteroids	Ninety-four percent of patients had an initial response (77% complete response and 16% partial response). No infusional toxicities or ectopic tissue formations were reported.
2010 [17]	11	Unrelated HLA disparate donors	Median dose was 1.2 x 10(6)/kg (range: 0.7-3.7 x 10(6)/kg).	Overall response was 71.4%, with complete response in 23.8% of cases. None patients presented GVHD progression upon MSC administration, but 4 patients presented GVHD recurrence 2 to 5 months after infusion. Two patients developed chronic limited GVHD.
2011 [18]	12	premanufactured, universal donor	8 x 10(6)cells/kg in 2 patients and 2 x 10(6)cells/kg in the rest	7 (58%) patients had complete response, 2 (17%) partial response, and 3 (25%) mixed response. Complete resolution of GI symptoms occurred in 9 (75%) patients. The cumulative incidence of survival at 100 days from the initiation of therapy was 58%.

All these studies with varying numbers of patients and different degrees of GVHD severity suggest that complete and partial responses can be achieved in a majority of patients after MSCs infusion and that MSCs might represent a potential novel therapy for GVHD.

## MSCs for cardiovascular repair

Despite progression of treatment options, ischemic heart disease and congestive heart failure remain major causes of morbidity and mortality. Cellular therapy for cardiovascular disease heralds an exciting frontier of research. Among the used cell types, MSCs are an attractive candidate for cardiovascular repair due to their abovementioned biological properties. In preclinical studies using experimental animal models of cardiac injury, MSCs had been show to engraft after systemic or local administration and improve the repair of infarcted myocardium [19-21]. In a rat model of dilated cardiomyopathy, Nagaya N et al found that MSC transplantation significantly increased capillary density and decreased the collagen volume fraction in the myocardium, resulting in decreased left ventricular end-diastolic pressureand increased left ventricular maximum [21].

Clinical trials using MSCs to improve cardiac function have also demonstrated encouraging results. For instance, in a pilot study, sixty-nine patients who underwent primary percutaneous coronary intervention within 12 hours after onset of acute myocardial infarction were randomized to receive intracoronary injection of autologous bone marrow mesenchymal stem cell or standard saline. Several imagining techniques demonstrated that MSCs significantly improved left ventricular function [22]. We conducted a clinical trial which recruited sixty-nine patients with acute myocardial infarction after percutaneous coronary intervention (PCI). They were randomly divided into intracoronary injection of MSCs (n = 34) and saline (n = 35) groups. Three months after MSC transplantation, left ventricular ejection fraction (LVEF) in MSCs group increased significantly compared with that of pre-implantation and that of the control group [23].

Here we summarized the currently completed clinical trials registered with clinicaltrials.gov that using MSC to treat cardiovascular diseases (Table 2). While a number of studies demonstrated the therapeutic effects of MSC transplantation, the underlying mechanisms remain unclear. The beneficial effects of MSCs might be mediated not only by their differentiation into cardiomyocytes but also by their ability to secret large amounts of bioactive molecules.

**Table 2:** Completed clinical trials at present time with MSC expanded in vitro (http: //clinic altrials.gov)

**Condition**	**Patients (N)**	**MSC source**	**Delivery route**	**Phase**	**Study design**	**ClinicalTrials.gov identifier**
Myocardial Ischemia	31	Autologous MSC from bone marrow	intramyocardial injections	Phase I/II	Non-randomized, Single group assignment, Open label	NCT00260338
Acute Myocardial Infarction	80	Autologous MSC from bone marrow	intracoronary injection	Phase II/ III	Randomized, Parallel assignment, Open Label	NCT01392105
Ischemic Heart Disease	48	MSC from bone marrow	intracoronary injection	Phase I/II	Non-Randomized, Parallel Assignment, Open label	NCT00135850
Heart Failure	10	Not mentioned	intramyocardial injections	Phase II	Randomized, Parallel Assignment, Double blind (Subject, Caregiver, Investigator)	NCT00927784

## MSCs for liver disease

In regard to liver diseases, MSCs have been used to treat cirrhosis in a limited number of patients. In a phase I trial, four patients with decompensated liver cirrhosis were included. They received autologous MSC infusion through a peripheral vein. There were no side-effects in the patients during follow-up. The quality of life of all four patients improved by the end of follow-up [24]. In another phase I-II clinical trial, 8 patients (four hepatitis B, one hepatitis C, one alcoholic, and two cryptogenic) with end-stage liver disease were included. After autologous MSCs injection, all patients tolerated well and their liver function improved, suggesting the feasibility, safety, and efficacy of using MSCs as a treatment for end-stage liver disease [25]. To test the safety and efficacy of allogenic MSCs for Patients with refractory primary biliary cirrhosis (PBC), we are conducting an open-label, multiple centers, randomized, Phase I-II clinical trial (ClinicalTrials.gov ID NCT01440309).

## Biological characteristics of MSCs associated with their therapeutic effects

**Figure 3:**
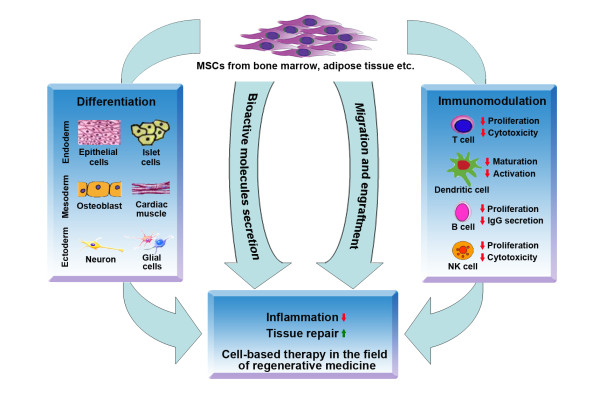
A schematic model demonstrating the biological properties of MSCs that are associated with their therapeutic effects.

The use of MSCs in clinical applications requires understanding of their biological characteristics that contribute to the therapeutic effects. Currently, the following four properties are considered the most important (Figure 3): (1) the ability to home to sites of inflammation following tissue injury when injected intravenously (2) the ability to differentiate into various cell types (3) the ability to secrete multiple bioactive molecules capable of stimulating recovery of injured cells and inhibiting inflammation (4) the lack of immunogenicity and the ability to perform immunomodulatory functions. Although we divide the effects of MSCs into these four aspects for better description in this review, in fact, these four aspects are combined and overlapped. Their exact roles in the therapeutic effects of MSCs remain to be further elucidated.

## Capacity to migrate and engraft

MSCs have the capacity to migrate to, and engraft in, sites of inflammation after systematic administration and exert local, functional effects in the resident tissue. Various studies have demonstrated that under a variety of pathologic conditions, MSC selectively home to sites of injury, irrespective of the tissue. Ortiz LA et al showed that murine MSCs could home to lung in response to injury, adopt an epithelium-like phenotype, and reduce inflammation in lung tissue of mice challenged with bleomycin [26]. We found that transplanted MSCs could migrate to injured muscle tissues in mdx mice [27].

Cell migration is dependent on a multitude of signals ranging from growth factors to chemokines secreted by injured cells and/or respondent immune cells [28]. Migration of MSCs may also be regulated by such signals. Studies have demonstrated that MSCs migration is under the control of a large range of receptor tyrosine kinase growth factors such as platelet-derived growth factor (PDGF) or insulin-like growth factor 1 (IGF-1) and chemokines such as CCR2, CCR3, CCR4 or CCL5 as assessed by in vitro migration assays [29].

## Differentiation

MSCs have the capacity to differentiate into mesenchymal lineages including osteoblasts, adipocytes, and chondroblasts under both in vitro and in vivo conditions [30]. Studies have also reported that MSCs can give rise to cells of other lineages. We found that MSC injected immediately into C57BL/6 mice after irradiation-caused injury could differentiate into functional lung cells, such as epithelial and endothelial cells [31]. Other studies employing animal model of lung injury caused by bleomycin exposure showed that MSCs engrafted in lung differentiated into type I pneumocytes [32] and type II epithelial cells [26] or assumed phenotypic characteristics of all major cell types in lung including fibroblasts, epithelial cells, and myofibroblasts [33]. In addition, MSCs could be induced to differentiate into cells of ectoderm. For example, Kopen GC et al were the first to demonstrate that MSCs injected into the central nervous systems of newborn mice could adopt morphological and phenotypic characteristics of astrocytes and neurons [34]. Subsequent studies confirmed such findings [35,36].

The evidence that MSCs were able to differentiate into specialized cells of tissues such as epithelial cells or nervous cells, opened up the possibility of using MSCs to substitute damaged cells for disease treatment. We showed that in a C57BL/6 mouse model of ischemia-reperfusion (I/R) kidney, transplanted MSCs were able to differentiate toward renal tubular epithelium at an early stage of injury. The differentiated donor cells replaced the vacant space left over by the dead cells, therefore contributing to the maintenance of structural integrity and preceded to a subsequent tissue repair process [37]. Several studies also demonstrated the contribution of MSCs differentiation to disease treatment [27]. However, accumulating data suggest the replacement of the damaged cells by MSCs through specific differentiation may be only a small part of the mechanism underlying MSCs’ therapeutic effects.

## Secreting multiple bioactive molecules

MSCs could secrete multiple bioactive molecules including many known growth factors, cytokines and chemokines which have profound effects on local cellular dynamics (Table 3). Administration of conditioned medium of MSCs is able to recapitulate the beneficial effects of MSCs for tissue repair. For instance, data from Van Poll D et al provide the first clear evidence that MSCs conditioned medium (MSC-CM) provides trophic support to the injured liver by inhibiting hepatocellular death and stimulating regeneration, potentially creating new avenues for the treatment of fulminant hepatic failure (FHF) [38]. Takahashi M et al demonstrated that various cytokines were produced by BM-MSCs, and these cytokines contributed to functional improvement of the infarcted heart by directly preserving the contractile capacity of the myocardium, inhibiting apoptosis of cardiomyocytes, and inducing therapeutic angiogenesis of the infarcted heart [39].

**Table 3:** Important bioactive molecules secreted by MSCs and their functions

**Bioactive molecules**	**Functions**
prostaglandin-E2 (PGE2)	anti-proliferative mediators [40]
	anti-inflammation [41]
Interleukin-10(IL-10)	anti-inflammatory [42,43]
transforming growth	suppress T-lymphocyte proliferation [44]
factorβ-1(TGFβ1), hepatocyte growth factor(HGF)	
Interleukin-1 receptor Antagonist	anti-inflammatory [45]
human leukocyte antigen G isoform (HLA-G5)	anti-proliferative for naive
	T-cells [46]
LL-37	anti-microbial peptide and reduce inflammation [47]
angiopoietin-1	restore epithelial protein permeability [48]
MMP3, MMP9	mediating neovascularization [49]
Keratinocyte growth factor	Alveolar epithelial fluid transport [50]
endothelial growth factor (VEGF), basic fibroblast growth factor (bFGF), placental growth factor (PlGF), and monocyte chemoattractant protein-1 (MCP-1)	enhance proliferation of endothelial cells and smooth muscle cells [51,52]

A protein-array analysis of MSC-CM detected 69 of 174 assayed proteins and most of these detected molecules are growth factors, cytokines, and chemokines. They have known anti-apoptotic and regeneration-stimulating effects [53]. These effects can be either direct or indirect or both: direct by causing intracellular signaling or indirect by causing another cell in the microenvironment to secrete functionally active agent.

## Immunomodulatory functions of MSCs

The ability of MSCs to modulate the immune system was first recognized in 2000 when Liechty KW et al found that MSCs have unique immunologic characteristics that allow their persistence in a xenogeneic environment [54]. Since then, an emerging body of data confirmed the immunomodulatory properties of MSCs. However, the precise mechanisms underlying their immunomodulation are still not fully understood. Direct cell-to-cell contact and/or release of soluble immunosuppressive factors may play major roles.

MSCs could interact with a wide range of immune cells, including T lymphocytes, B lymphocytes, natural killer cells and dendritic cells. A brief summary of the in vitro interaction of MSCs and immune cells was shown in Table 4. The immunomodulatory effects of MSCs have also been examined in a variety of animal models of immune diseases. For instance, donor-derived MSC could induce long-term allograft acceptance in a rat heart transplantation model [55] .

**Table 4:** Immunomodulatory effects of MSCs on immune cells

**Immune cell type**	**MSCs’ effects**
T lymphocyte	Suppress T cell proliferation induced by cellular or nonspecific mitogenic stimuli [44]
	Alter the cytokine secretion profile of naive and effector T cells [56]
	Promote the expansion and function of Treg cells [57]
B lymphocyte	Inhibit proliferation of B lymphocyte [58]
	Affect the chemotactic properties of B cells [59]
	Suppress B-cell terminal differentiation [60]
NK cell	Alter the phenotype of NK cells and suppress proliferation, cytokine secretion, and cyto-toxicity against HLA-class I- expressing targets [61,62]
Dendritic cells (DCs)	Influence differentiation, maturation and function of monocyte-derived dendritic cells [63]
	Suppress dendritic cell migration, maturation and antigen presentation [64]
	Induce mature DCs into a novel Jagged-2-dependent regulatory DC population [65]

The immunomodulatory functions of MSCs have generated a great amount of interest in their potential for treatment of immune disorders such as GVHD.

## Discussion and future directions

Over the past decade, there have been a large number of publications on MSCs, reporting their biological properties, experimental and clinical applications or underlying molecular mechanisms. Although tremendous advancements have been made from both preclinical and clinical studies using MSCs, substantial challenges are still to be overcome before MSC therapy can fulfill its promise in wider clinical practice. (1) Safety issue: up to now, few adverse effects have been reported after MSC administration, in terms of immediate, infusional toxicity and of late effects. However, the relatively small number of patients being treated with MSCs does not allow the drawing of definitive conclusions on the safety of MSCs. Furthermore, MSCs has been reported to promote tumor growth [66] and metastases [67]. Potential for malignant transformation of cultured MSC commonly used in clinical cell-therapy protocols has also been reviewed [68]. In addition, under some pathological conditions, application of MSCs might do more harm than good. We found that MSCs could aggravate arthritis in collagen-induced arthritis model by at least up-regulating secretion of IL-6, which favors Th17 differentiation [69]. These studies remind us that particular attention should be paid to the biosafety of MSC. (2) Quality control: Cell amplification by culture is not free from the dangers of microbial contamination, thus bacteriological tests (mainly in liquid medium) should be carefully performed during the various phases of production and at harvest. In addition, viability and phenotype tests, oncogenicity tests and endotoxin assay should also be included. In addition, optimal timing of MSC administration, cell dose and schedule of administration need to be defined according to disease types and severity. (3)Clinical grade production:Clinical application of MSC requires a large number of cells for transplantation, so in vitro expansion of MSC is inevitable. Studies have suggested that continuous passaging of MSCs could lead to cell transformation. Rubio D et al found that human mesenchymal stem cells could undergo spontaneous transformation following long-term in vitro culture (4–5 months).The transformed cells exhibited chromosomal abnormalities, increased c-myc levels and telomerase activity, and formed tumours on transplantation [70]. To reduce malignant transformation of human MSCs, meticulous attention must be taken to prevent cell senescence and limit the number of passaging. According to Bernardo ME et al [71], MSCs can be safely expanded in vitro until passage 25. We conducted the first stem cell clinical trial approved from SFDA in China and our standard procedure requires that the optimal passage should be less than passages 6 during the manufacture of MSC. MSCs used in clinical trials must be manufactured under the conditions required by Good Manufacturing Practice (GMP). (4) Autologous vs allogeneic MSCs: MSC are immune privileged because they express low levels of major histocompatibility complex-I (MHC-I) molecules and do not express MHCII molecules or costimulatory molecules such as CD80, CD86 or CD40 [15]. This unique property allows for the transplantation of allogeneic MSCs without inducing immune rejection. Thus both autologous and allogeneic MSCs can be used in the clinical setting. However, which one to prefer needs further investigation. (5) Clinical transition: In the field of MSCs research, biologists and clinicians should come together to establish proper and stringent regulations and standards for MSC based therapies. The regulations and standards should at least include methods and criteria for the culture, storage, shipping, and administration of MSCs.

MSC therapies are undergoing rapid development and have generated great excitement amongst scientists and physicians. Currently, more randomized, controlled, multi-centre clinical trials are needed to find the optimal conditions for MSC therapy. We believe that eventually a novel and safe therapy with MSCs can emerge and revolutionize treatment and therapies for patients with severe diseases.

## **Abbreviations**

MS, Mesenchymal stem cells; GVHD, Graft-versus-host-disease; ESC, Embryonic stem cell; iPSC, Pluripotent stem cell; AMI, Acute myocardial ischemia; ALS, Amyotrophic lateral sclerosis; aGVHD, Acute graft-versus-host disease; PCI, Percutaneous coronary intervention; LVEF, Left ventricular ejection fraction; PBC, Primary biliary cirrhosis; PDGF, Platelet-derived growth factor; IGF-1, Insulin-like growth factor 1; FHF, Fulminant hepatic failure; DCs, Dendritic cells; GMP, Good manufacturing practice.

## Competing interests

We declare that we have no competing interests.

## Authors’ contributions

SW drafted the manuscript; XQ formatted the figures and tables, participated in manuscript revision; RCZ designed and drafted the manuscript, gave financial and administrative support. All authors read and approved the final manuscript.

## References

[B1] Friedenstein AJ Petrakova KV Kurolesova AI Frolova GP Heterotopic of bone marrow. Analysis of precursor cells for osteogenic and hematopoietic tissues Transplantation 1968 6 230 247 10.1097/00007890-196803000-00009 5654088

[B2] In 't Anker PS Scherjon SA Kleijburg-van Dder Keur C de Groot-Swings GM Claas FH Fibbe WE Kanhai HH Isolation of mesenchymal stem cells of fetal or maternal origin from human placenta Stem Cells 2004 22 1338 1345 10.1634/stemcells.2004-0058 15579651

[B3] Dominici M Le Blanc K Mueller I Slaper-Cortenbach I Marini F Krause D Deans R Keating A Prockop D Horwitz E Minimal criteria for defining multipotent mesenchymal stromal cells The International Society for Cellular Therapy position statement. Cytotherapy 2006 8 315 317 10.1080/14653240600855905 16923606

[B4] Lazarus HM Haynesworth SE Gerson SL Rosenthal NS Caplan AI Ex vivo expansion and subsequent infusion of human bone marrow-derived stromal progenitor cells (mesenchymal progenitor cells): implications for therapeutic use Bone Marrow Transplant 1995 16 557 564 8528172

[B5] Otto WR Wright NA Mesenchymal stem cells: from experiment to clinic Fibrogenesis Tissue Repair 2011 4 20 10.1186/1755-1536-4-20 21902837 PMC3182886

[B6] Messina C Faraci M de Fazio V Dini G Calo MP Calore E Prevention and treatment of acute GvHD Bone Marrow Transplant 2008 41 Suppl 2 S65 70 18545247 10.1038/bmt.2008.57

[B7] Sun ZM Liu HL Geng LQ Wang XB Yao W Liu X Ding KY Han YS Yang HZ Tang BL HLA-matched sibling transplantation with G-CSF mobilized PBSCs and BM decreases GVHD in adult patients with severe aplastic anemia J Hematol Oncol 2010 3 51 10.1186/1756-8722-3-51 21194460 PMC3023734

[B8] Huang XJ Current status of haploidentical stem cell transplantation for leukemia J Hematol Oncol 2008 1 27 10.1186/1756-8722-1-27 19117511 PMC2637880

[B9] Le Blanc K Rasmusson I Sundberg B Gotherstrom C Hassan M Uzunel M Ringden O Treatment of severe acute graft-versus-host disease with third party haploidentical mesenchymal stem cells Lancet 2004 363 1439 1441 10.1016/S0140-6736(04)16104-7 15121408

[B10] Ringden O Uzunel M Rasmusson I Remberger M Sundberg B Lonnies H Marschall HU Dlugosz A Szakos A Hassan Z Mesenchymal stem cells for treatment of therapy-resistant graft-versus-host disease Transplantation 2006 81 1390 1397 10.1097/01.tp.0000214462.63943.14 16732175

[B11] Fang B Song Y Liao L Zhang Y Zhao RC Favorable response to human adipose tissue-derived mesenchymal stem cells in steroid-refractory acute graft-versus-host disease Transplant Proc 2007 39 3358 3362 10.1016/j.transproceed.2007.08.103 18089385

[B12] Le Blanc K Frassoni F Ball L Locatelli F Roelofs H Lewis I Lanino E Sundberg B Bernardo ME Remberger M Mesenchymal stem cells for treatment of steroid-resistant, severe, acute graft-versus-host disease: a phase II study Lancet 2008 371 1579 1586 10.1016/S0140-6736(08)60690-X 18468541

[B13] Muller I Kordowich S Holzwarth C Isensee G Lang P Neunhoeffer F Dominici M Greil J Handgretinger R Application of multipotent mesenchymal stromal cells in pediatric patients following allogeneic stem cell transplantation Blood Cells Mol Dis 2008 40 25 32 10.1016/j.bcmd.2007.06.021 17869550

[B14] von Bonin M Stolzel F Goedecke A Richter K Wuschek N Holig K Platzbecker U Illmer T Schaich M Schetelig J Treatment of refractory acute GVHD with third-party MSC expanded in platelet lysate-containing medium Bone Marrow Transplant 2009 43 245 251 10.1038/bmt.2008.316 18820709

[B15] Guo M Sun Z Sun QY Han Q Yu CL Wang DH Qiao JH Chen B Sun WJ Hu KX A modified haploidentical nonmyeloablative transplantation without T cell depletion for high-risk acute leukemia: successful engraftment and mild GVHD Biol Blood Marrow Transplant 2009 15 930 937 10.1016/j.bbmt.2009.04.006 19589482

[B16] Kebriaei P Isola L Bahceci E Holland K Rowley S McGuirk J Devetten M Jansen J Herzig R Schuster M Adult human mesenchymal stem cells added to corticosteroid therapy for the treatment of acute graft-versus-host disease Biol Blood Marrow Transplant 2009 15 804 811 10.1016/j.bbmt.2008.03.012 19539211

[B17] Lucchini G Introna M Dander E Rovelli A Balduzzi A Bonanomi S Salvade A Capelli C Belotti D Gaipa G Platelet-lysate-expanded mesenchymal stromal cells as a salvage therapy for severe resistant graft-versus-host disease in a pediatric population Biol Blood Marrow Transplant 2010 16 1293 1301 10.1016/j.bbmt.2010.03.017 20350611

[B18] Prasad VK Lucas KG Kleiner GI Talano JA Jacobsohn D Broadwater G Monroy R Kurtzberg J Efficacy and safety of ex vivo cultured adult human mesenchymal stem cells (Prochymal) in pediatric patients with severe refractory acute graft-versus-host disease in a compassionate use study Biol Blood Marrow Transplant 2011 17 534 541 10.1016/j.bbmt.2010.04.014 20457269

[B19] Zhang S Ge J Sun A Xu D Qian J Lin J Zhao Y Hu H Li Y Wang K Comparison of various kinds of bone marrow stem cells for the repair of infarcted myocardium: single clonally purified non-hematopoietic mesenchymal stem cells serve as a superior source J Cell Biochem 2006 99 1132 1147 10.1002/jcb.20949 16795039

[B20] Jiang S Haider H Idris NM Salim A Ashraf M Supportive interaction between cell survival signaling and angiocompetent factors enhances donor cell survival and promotes angiomyogenesis for cardiac repair Circ Res 2006 99 776 784 10.1161/01.RES.0000244687.97719.4f 16960098

[B21] Nagaya N Kangawa K Itoh T Iwase T Murakami S Miyahara Y Fujii T Uematsu M Ohgushi H Yamagishi M Transplantation of mesenchymal stem cells improves cardiac function in a rat model of dilated cardiomyopathy Circulation 2005 112 1128 1135 10.1161/CIRCULATIONAHA.104.500447 16103243

[B22] Chen SL Fang WW Ye F Liu YH Qian J Shan SJ Zhang JJ Chunhua RZ Liao LM Lin S Effect on left ventricular function of intracoronary transplantation of autologous bone marrow mesenchymal stem cell in patients with acute myocardial infarction Am J Cardiol 2004 94 92 95 10.1016/j.amjcard.2004.03.034 15219514

[B23] Chen SL Fang WW Qian J Ye F Liu YH Shan SJ Zhang JJ Lin S Liao LM Zhao RC Improvement of cardiac function after transplantation of autologous bone marrow mesenchymal stem cells in patients with acute myocardial infarction Chin Med J (Engl) 2004 117 1443 1448 15498362

[B24] Mohamadnejad M Alimoghaddam K Mohyeddin-Bonab M Bagheri M Bashtar M Ghanaati H Baharvand H Ghavamzadeh A Malekzadeh R Phase 1 trial of autologous bone marrow mesenchymal stem cell transplantation in patients with decompensated liver cirrhosis Arch Iran Med 2007 10 459 466 17903050

[B25] Kharaziha P Hellstrom PM Noorinayer B Farzaneh F Aghajani K Jafari F Telkabadi M Atashi A Honardoost M Zali MR Improvement of liver function in liver cirrhosis patients after autologous mesenchymal stem cell injection: a phase I-II clinical trial Eur J Gastroenterol Hepatol 2009 21 1199 1205 10.1097/MEG.0b013e32832a1f6c 19455046

[B26] Ortiz LA Gambelli F McBride C Gaupp D Baddoo M Kaminski N Phinney DG Mesenchymal stem cell engraftment in lung is enhanced in response to bleomycin exposure and ameliorates its fibrotic effects Proc Natl Acad Sci U S A 2003 100 8407 8411 10.1073/pnas.1432929100 12815096 PMC166242

[B27] Liu Y Yan X Sun Z Chen B Han Q Li J Zhao RC Flk-1+ adipose-derived mesenchymal stem cells differentiate into skeletal muscle satellite cells and ameliorate muscular dystrophy in mdx mice Stem Cells Dev 2007 16 695 706 10.1089/scd.2006.0118 17999592

[B28] Spaeth E Klopp A Dembinski J Andreeff M Marini F Inflammation and tumor microenvironments: defining the migratory itinerary of mesenchymal stem cells Gene Ther 2008 15 730 738 10.1038/gt.2008.39 18401438

[B29] Yagi H Soto-Gutierrez A Parekkadan B Kitagawa Y Tompkins RG Kobayashi N Yarmush ML Mesenchymal stem cells: Mechanisms of immunomodulation and homing Cell Transplant 2010 19 667 679 10.3727/096368910X508762 20525442 PMC2957533

[B30] Dan YY Riehle KJ Lazaro C Teoh N Haque J Campbell JS Fausto N Isolation of multipotent progenitor cells from human fetal liver capable of differentiating into liver and mesenchymal lineages Proc Natl Acad Sci U S A 2006 103 9912 9917 10.1073/pnas.0603824103 16782807 PMC1502553

[B31] Yan X Liu Y Han Q Jia M Liao L Qi M Zhao RC Injured microenvironment directly guides the differentiation of engrafted Flk-1(+) mesenchymal stem cell in lung Exp Hematol 2007 35 1466 1475 10.1016/j.exphem.2007.05.012 17637496

[B32] Kotton DN Ma BY Cardoso WV Sanderson EA Summer RS Williams MC Fine A Bone marrow-derived cells as progenitors of lung alveolar epithelium Development 2001 128 5181 5188 11748153 10.1242/dev.128.24.5181

[B33] Rojas M Xu J Woods CR Mora AL Spears W Roman J Brigham KL Bone marrow-derived mesenchymal stem cells in repair of the injured lung Am J Respir Cell Mol Biol 2005 33 145 152 10.1165/rcmb.2004-0330OC 15891110 PMC2715309

[B34] Kopen GC Prockop DJ Phinney DG Marrow stromal cells migrate throughout forebrain and cerebellum, and they differentiate into astrocytes after injection into neonatal mouse brains Proc Natl Acad Sci U S A 1999 96 10711 10716 10.1073/pnas.96.19.10711 10485891 PMC17948

[B35] Deng J Petersen BE Steindler DA Jorgensen ML Laywell ED Mesenchymal stem cells spontaneously express neural proteins in culture and are neurogenic after transplantation Stem Cells 2006 24 1054 1064 10.1634/stemcells.2005-0370 16322639

[B36] Wislet-Gendebien S Hans G Leprince P Rigo JM Moonen G Rogister B Plasticity of cultured mesenchymal stem cells: switch from nestin-positive to excitable neuron-like phenotype Stem Cells 2005 23 392 402 10.1634/stemcells.2004-0149 15749934

[B37] Li K Han Q Yan X Liao L Zhao RC Not a process of simple vicariousness, the differentiation of human adipose-derived mesenchymal stem cells to renal tubular epithelial cells plays an important role in acute kidney injury repairing Stem Cells Dev 2010 19 1267 1275 10.1089/scd.2009.0196 19874085

[B38] van Poll D Parekkadan B Cho CH Berthiaume F Nahmias Y Tilles AW Yarmush ML Mesenchymal stem cell-derived molecules directly modulate hepatocellular death and regeneration in vitro and in vivo Hepatology 2008 47 1634 1643 10.1002/hep.22236 18395843

[B39] Takahashi M Li TS Suzuki R Kobayashi T Ito H Ikeda Y Matsuzaki M Hamano K Cytokines produced by bone marrow cells can contribute to functional improvement of the infarcted heart by protecting cardiomyocytes from ischemic injury Am J Physiol Heart Circ Physiol 2006 291 H886 893 10.1152/ajpheart.00142.2006 16603697

[B40] Bouffi C Bony C Courties G Jorgensen C Noel D IL-6-dependent PGE2 secretion by mesenchymal stem cells inhibits local inflammation in experimental arthritis PLoS One 2010 5 e14247 10.1371/journal.pone.0014247 21151872 PMC2998425

[B41] Foraker JE Oh JY Ylostalo JH Lee RH Watanabe J Prockop DJ Cross-talk between human mesenchymal stem/progenitor cells (MSCs) and rat hippocampal slices in LPS-stimulated cocultures: the MSCs are activated to secrete prostaglandin E2 J Neurochem 2011 119 1052 1063 10.1111/j.1471-4159.2011.07511.x 21954847

[B42] Nemeth K Leelahavanichkul A Yuen PS Mayer B Parmelee A Doi K Robey PG Leelahavanichkul K Koller BH Brown JM Bone marrow stromal cells attenuate sepsis via prostaglandin E(2)-dependent reprogramming of host macrophages to increase their interleukin-10 production Nat Med 2009 15 42 49 10.1038/nm.1905 19098906 PMC2706487

[B43] Gupta N Su X Popov B Lee JW Serikov V Matthay MA Intrapulmonary delivery of bone marrow-derived mesenchymal stem cells improves survival and attenuates endotoxin-induced acute lung injury in mice J Immunol 2007 179 1855 1863 17641052 10.4049/jimmunol.179.3.1855

[B44] Di Nicola M Carlo-Stella C Magni M Milanesi M Longoni PD Matteucci P Grisanti S Gianni AM Human bone marrow stromal cells suppress T-lymphocyte proliferation induced by cellular or nonspecific mitogenic stimuli Blood 2002 99 3838 3843 10.1182/blood.V99.10.3838 11986244

[B45] Ortiz LA Dutreil M Fattman C Pandey AC Torres G Go K Phinney DG Interleukin 1 receptor antagonist mediates the antiinflammatory and antifibrotic effect of mesenchymal stem cells during lung injury Proc Natl Acad Sci U S A 2007 104 11002 11007 10.1073/pnas.0704421104 17569781 PMC1891813

[B46] Selmani Z Naji A Zidi I Favier B Gaiffe E Obert L Borg C Saas P Tiberghien P Rouas-Freiss N Human leukocyte antigen-G5 secretion by human mesenchymal stem cells is required to suppress T lymphocyte and natural killer function and to induce CD4 + CD25highFOXP3+ regulatory T cells Stem Cells 2008 26 212 222 10.1634/stemcells.2007-0554 17932417

[B47] Krasnodembskaya A Song Y Fang X Gupta N Serikov V Lee JW Matthay MA Antibacterial effect of human mesenchymal stem cells is mediated in part from secretion of the antimicrobial peptide LL-37 Stem Cells 2010 28 2229 2238 10.1002/stem.544 20945332 PMC3293245

[B48] Fang X Neyrinck AP Matthay MA Lee JW Allogeneic human mesenchymal stem cells restore epithelial protein permeability in cultured human alveolar type II cells by secretion of angiopoietin-1 J Biol Chem 2010 285 26211 26222 10.1074/jbc.M110.119917 20554518 PMC2924032

[B49] Kim Y Kim H Cho H Bae Y Suh K Jung J Direct comparison of human mesenchymal stem cells derived from adipose tissues and bone marrow in mediating neovascularization in response to vascular ischemia Cell Physiol Biochem 2007 20 867 876 10.1159/000110447 17982269

[B50] Lee JW Fang X Gupta N Serikov V Matthay MA Allogeneic human mesenchymal stem cells for treatment of E. coli endotoxin-induced acute lung injury in the ex vivo perfused human lung Proc Natl Acad Sci U S A 2009 106 16357 16362 10.1073/pnas.0907996106 19721001 PMC2735560

[B51] Kinnaird T Stabile E Burnett MS Shou M Lee CW Barr S Fuchs S Epstein SE Local delivery of marrow-derived stromal cells augments collateral perfusion through paracrine mechanisms Circulation 2004 109 1543 1549 10.1161/01.CIR.0000124062.31102.57 15023891

[B52] Kinnaird T Stabile E Burnett MS Lee CW Barr S Fuchs S Epstein SE Marrow-derived stromal cells express genes encoding a broad spectrum of arteriogenic cytokines and promote in vitro and in vivo arteriogenesis through paracrine mechanisms Circ Res 2004 94 678 685 10.1161/01.RES.0000118601.37875.AC 14739163

[B53] Parekkadan B van Poll D Suganuma K Carter EA Berthiaume F Tilles AW Yarmush ML Mesenchymal stem cell-derived molecules reverse fulminant hepatic failure PLoS One 2007 2 e941 10.1371/journal.pone.0000941 17895982 PMC1978513

[B54] Liechty KW MacKenzie TC Shaaban AF Radu A Moseley AM Deans R Marshak DR Flake AW Human mesenchymal stem cells engraft and demonstrate site-specific differentiation after in utero transplantation in sheep Nat Med 2000 6 1282 1286 10.1038/81395 11062543

[B55] Popp FC Eggenhofer E Renner P Slowik P Lang SA Kaspar H Geissler EK Piso P Schlitt HJ Dahlke MH Mesenchymal stem cells can induce long-term acceptance of solid organ allografts in synergy with low-dose mycophenolate Transpl Immunol 2008 20 55 60 10.1016/j.trim.2008.08.004 18762258

[B56] Aggarwal S Pittenger MF Human mesenchymal stem cells modulate allogeneic immune cell responses Blood 2005 105 1815 1822 10.1182/blood-2004-04-1559 15494428

[B57] English K Ryan JM Tobin L Murphy MJ Barry FP Mahon BP Cell contact, prostaglandin E(2) and transforming growth factor beta 1 play non-redundant roles in human mesenchymal stem cell induction of CD4 + CD25(High) forkhead box P3+ regulatory T cells Clin Exp Immunol 2009 156 149 160 10.1111/j.1365-2249.2009.03874.x 19210524 PMC2673753

[B58] Augello A Tasso R Negrini SM Amateis A Indiveri F Cancedda R Pennesi G Bone marrow mesenchymal progenitor cells inhibit lymphocyte proliferation by activation of the programmed death 1 pathway Eur J Immunol 2005 35 1482 1490 10.1002/eji.200425405 15827960

[B59] Corcione A Benvenuto F Ferretti E Giunti D Cappiello V Cazzanti F Risso M Gualandi F Mancardi GL Pistoia V Human mesenchymal stem cells modulate B-cell functions Blood 2006 107 367 372 10.1182/blood-2005-07-2657 16141348

[B60] Asari S Itakura S Ferreri K Liu CP Kuroda Y Kandeel F Mullen Y Mesenchymal stem cells suppress B-cell terminal differentiation Exp Hematol 2009 37 604 615 10.1016/j.exphem.2009.01.005 19375651 PMC2747661

[B61] Sotiropoulou PA Perez SA Gritzapis AD Baxevanis CN Papamichail M Interactions between human mesenchymal stem cells and natural killer cells Stem Cells 2006 24 74 85 10.1634/stemcells.2004-0359 16099998

[B62] Spaggiari GM Capobianco A Becchetti S Mingari MC Moretta L Mesenchymal stem cell-natural killer cell interactions: evidence that activated NK cells are capable of killing MSCs, whereas MSCs can inhibit IL-2-induced NK-cell proliferation Blood 2006 107 1484 1490 10.1182/blood-2005-07-2775 16239427

[B63] Zhang W Ge W Li C You S Liao L Han Q Deng W Zhao RC Effects of mesenchymal stem cells on differentiation, maturation, and function of human monocyte-derived dendritic cells Stem Cells Dev 2004 13 263 271 10.1089/154732804323099190 15186722

[B64] Chen L Zhang W Yue H Han Q Chen B Shi M Li J Li B You S Shi Y Effects of human mesenchymal stem cells on the differentiation of dendritic cells from CD34+ cells Stem Cells Dev 2007 16 719 731 10.1089/scd.2007.0065 17999594

[B65] Zhang B Liu R Shi D Liu X Chen Y Dou X Zhu X Lu C Liang W Liao L Mesenchymal stem cells induce mature dendritic cells into a novel Jagged-2-dependent regulatory dendritic cell population Blood 2009 113 46 57 10.1182/blood-2008-04-154138 18832657

[B66] Djouad F Plence P Bony C Tropel P Apparailly F Sany J Noel D Jorgensen C Immunosuppressive effect of mesenchymal stem cells favors tumor growth in allogeneic animals Blood 2003 102 3837 3844 10.1182/blood-2003-04-1193 12881305

[B67] Karnoub AE Dash AB Vo AP Sullivan A Brooks MW Bell GW Richardson AL Polyak K Tubo R Weinberg RA Mesenchymal stem cells within tumour stroma promote breast cancer metastasis Nature 2007 449 557 563 10.1038/nature06188 17914389

[B68] Prockop DJ Brenner M Fibbe WE Horwitz E Le Blanc K Phinney DG Simmons PJ Sensebe L Keating A Defining the risks of mesenchymal stromal cell therapy Cytotherapy 2010 12 576 578 10.3109/14653249.2010.507330 20735162

[B69] Chen B Hu J Liao L Sun Z Han Q Song Z Zhao RC Flk-1+ mesenchymal stem cells aggravate collagen-induced arthritis by up-regulating interleukin-6 Clin Exp Immunol 2010 159 292 302 10.1111/j.1365-2249.2009.04069.x 20002448 PMC2819495

[B70] Rubio D Garcia-Castro J Martin MC de la Fuente R Cigudosa JC Lloyd AC Bernad A Spontaneous human adult stem cell transformation Cancer Res 2005 65 3035 3039 15833829 10.1158/0008-5472.CAN-04-4194

[B71] Bernardo ME Zaffaroni N Novara F Cometa AM Avanzini MA Moretta A Montagna D Maccario R Villa R Daidone MG Human bone marrow derived mesenchymal stem cells do not undergo transformation after long-term in vitro culture and do not exhibit telomere maintenance mechanisms Cancer Res 2007 67 9142 9149 10.1158/0008-5472.CAN-06-4690 17909019

